# Simultaneous diagnosis of familial achalasia: report of two cases

**DOI:** 10.1186/s40792-017-0340-0

**Published:** 2017-05-08

**Authors:** Masato Hoshino, Nobuo Omura, Fumiaki Yano, Se Ryung Yamamoto, Minoru Matsuda, Katsuhiko Yanaga

**Affiliations:** 1 Department of Surgery, Kasukabe Central General Hospital, 5-9-4 Midoricho, Kasukabe city, Saitama 344-0063 Japan; 2 Department of Surgery, Jikei University School of Medicine, 3-25-8 Nishishinbashi, Minato-ku, Tokyo, 105-8461 Japan

**Keywords:** Familial achalasia, High-resolution manometry (HRM), Genetic element

## Abstract

**Background:**

Achalasia is a rare disease with a morbidity of 1 in 100,000, for which the exact mechanism of pathogenesis has not been clarified due to the small total number of patients. We herein report on our experience with two cases of familial achalasia in which the involvement of genetic inheritance was suspected.

**Case presentation:**

These cases consist of a man in his thirties and his mother in her sixties. The son consulted the Department of Gastrointestinal Medicine at our institute with dysphagia, and an upper gastrointestinal endoscopy revealed a gastric submucosal tumor with a maximal diameter of approximately 50 mm. Achalasia was also strongly suspected due to the enlargement of the esophagus to the maximum transverse diameter of 55 mm by esophagography along with delayed clearance of barium. A detailed interview revealed prolonged mild dysphagia in his mother. Therefore, high-resolution manometry was carried out in both patients. As a result, peristaltic disorder was observed in the esophageal body in both the mother and son, leading to a definitive diagnosis of achalasia. For the son, total gastrectomy including the lower esophagus with Roux-en-Y reconstruction was performed. His postoperative course was uneventful, and the patient was discharged from hospital in remission on the 9th day following surgery and is currently undergoing follow-up as an outpatient.

**Conclusions:**

We hereby report on a very rare case of familial achalasia that we experienced which may suggest a genetic element in the onset of achalasia, and reviewed the literature.

## Background

Achalasia is a rare disease, with an annual incidence of 1 in 100,000 worldwide. Accordingly, the development of studies regarding its clinical condition is poor and the onset factors thereof are unknown [1, 2]. Achalasia is a disease characterized by impaired peristalsis of the esophageal body with relaxation failure of the lower esophageal sphincter (LES), lack of the first peristaltic wave of the esophagus, synchronized contraction, and histologically by the disappearance and degeneration of the Auerbach nerve plexus in the tunica muscularis esophagi; however, the cause of neurodegeneration is unknown. Currently, the involvement of viruses, immune disorders, genes, gastrointestinal hormones, and others have been implicated; however, the details thereof are unknown [3]. We hereby report on two cases we experienced in which a simultaneous diagnosis of familial achalasia was made.

## Case presentation

### Case 1

**Fig. 1:**
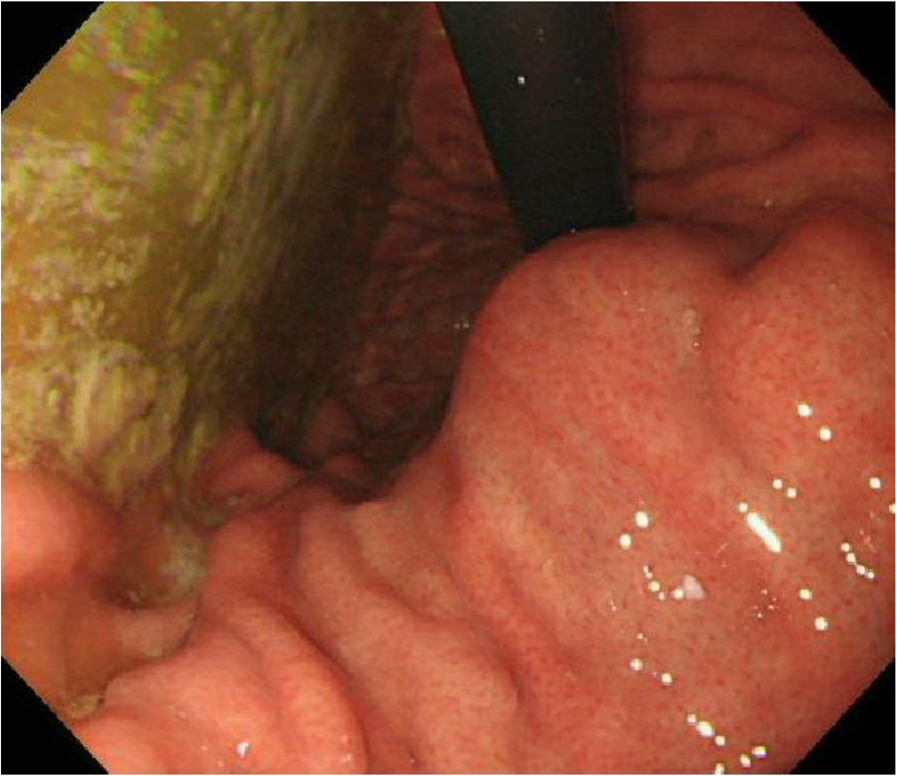
An inverted image inside the stomach upon upper gastrointestinal endoscopy. A submucosal tumor with a smooth surface having a maximal diameter of approximately 50 mm was observed in the lesser curvature of the upper corpus of the stomach

**Fig. 2:**
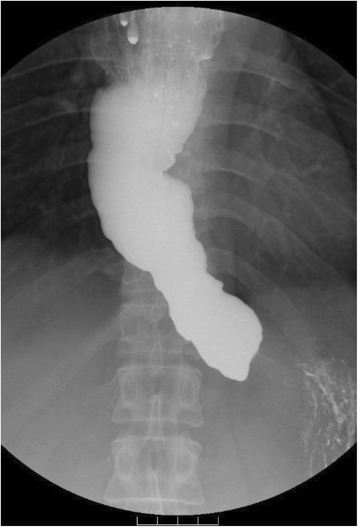
Images of the esophagography before surgery, which demonstrated esophageal dilatation with a maximum transverse diameter of 55 mm, which was classified as the *straight shape*

**Fig. 3:**
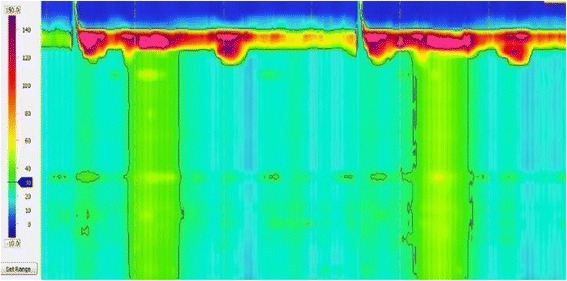
High-resolution manometry (HRM) findings before surgery in Case 1. Although the tip of the catheter could not to be placed inside the stomach beyond the LES, panesophageal presurrizations of esophageal body movement were recognized, which was classified as type 2 achalasia according to the Chicago classification

**Fig. 4:**
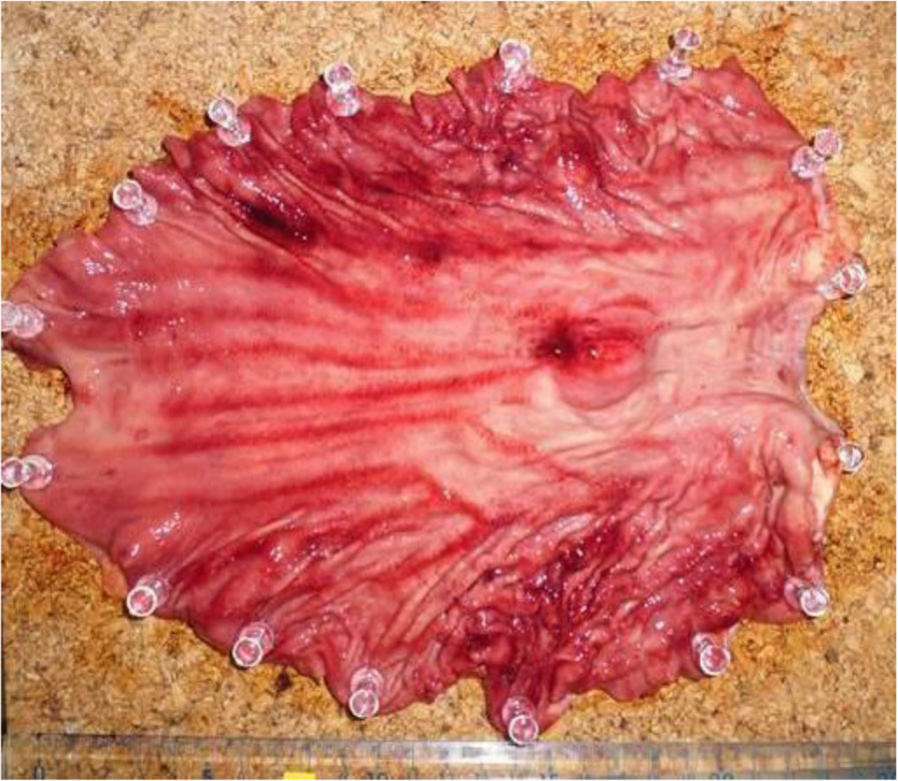
Macroscopic findings of the specimen. The proximal margin of the tumor was 30 mm from the EGJ

The case pertains to a man in his thirties with mental deficiency. He had an elder sister who had malignant colonic tumor (details unknown); however, she died a year ago. He consulted the Department of Gastrointestinal Medicine at our institute with dysphagia (the duration was 3 years) and no weight loss, and upper gastrointestinal endoscopy revealed a submucosal tumor with a maximal diameter of approximately 50 mm (Fig. 1). He underwent upper gastrointestinal series at our department, which revealed esophageal dilatation with a maximum transverse diameter of 55 mm, along with delayed esophageal clearance of barium, leading to the suspicion of achalasia (Fig. 2). High-resolution manometry (HRM) was carried out to make a definitive diagnosis, and a diagnosis of type 2 achalasia according to the Chicago classification was established (Fig. 3). A relatively well-defined, contrast-enhanced tumor with a maximal diameter of approximately 50 mm was observed in the lesser curvature of the upper gastric corpus by abdominal computed tomography (CT). Serum ACTH and cortisol were within normal ranges, and alacrima, muscular atrophy, and muscle weakness were not observed. From the findings mentioned above, the gastric submucosal tumor was diagnosed as a gastrointestinal stromal tumor (GIST) with achalasia, for which total gastrectomy including the lower esophagus was performed. Intraoperatively, an exophytic tumor was identified in the lesser curvature of the upper gastric corpus of the stomach, with multiple peritoneal metastatic nodules on the jejunal serosa. Despite non-curative, total gastrectomy with Roux-en-Y reconstruction was carried out to improve dysphagia and to avoid complications such as bleeding from the tumor (Fig. 4). A postoperative contrast study using gastrografin was carried out 4 days following surgery, in which passage from the esophagus to the jejunum was good without anastomotic leakage. The patient was discharged from the hospital on postoperative day 9 and is currently undergoing follow-up as an outpatient, with no dysphagia.

### Case 2

**Fig. 5:**
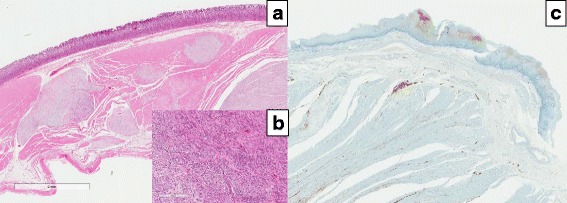
**a** Weak expansion and **b** strong expansion: macroscopic findings of the specimen (HE). Proliferation of spindle cells arranged in an interlacing pattern could be found. The gastric submucosal tumor was diagnosed as a GIST. **c** S-100 staining, it was decreasing in Case 1

**Fig. 6:**
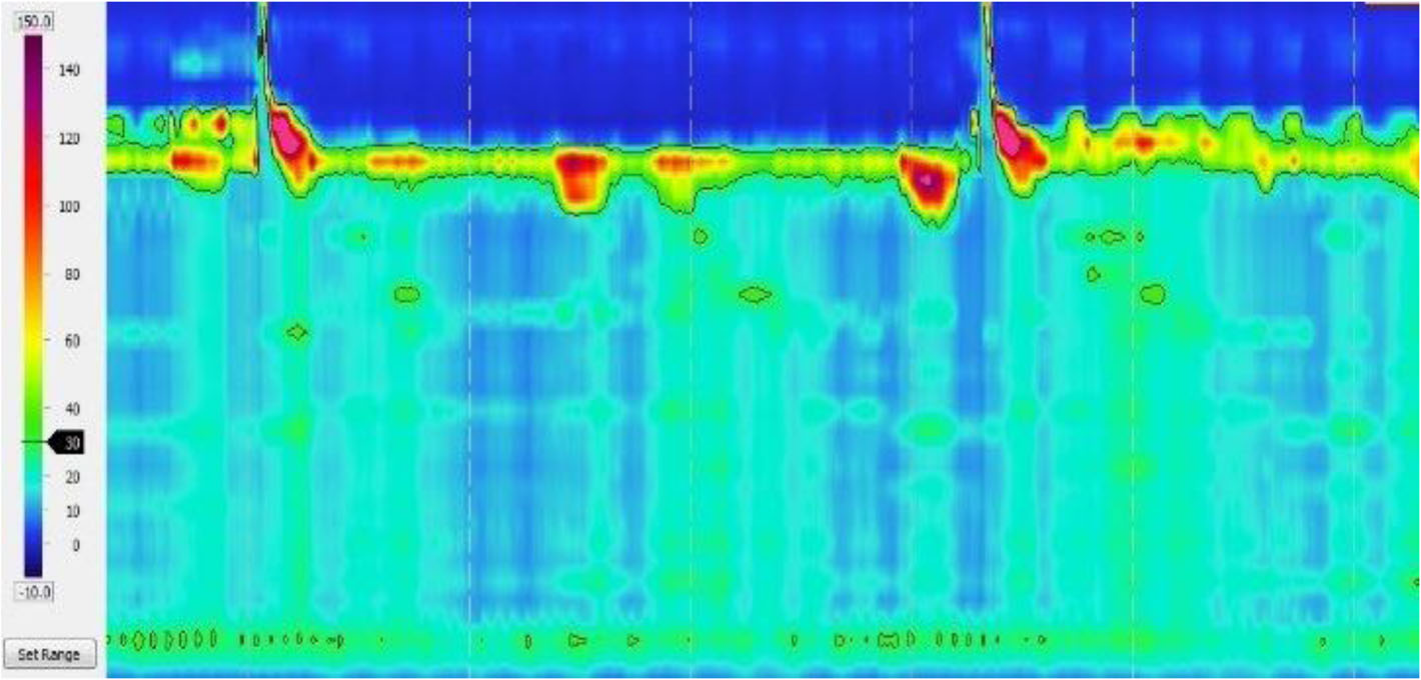
HRM findings in Case 2. IRP was 15.7 mmHg, and the esophageal body had no contractility and was classified as type 1 achalasia according to the Chicago classification

**Fig. 7:**
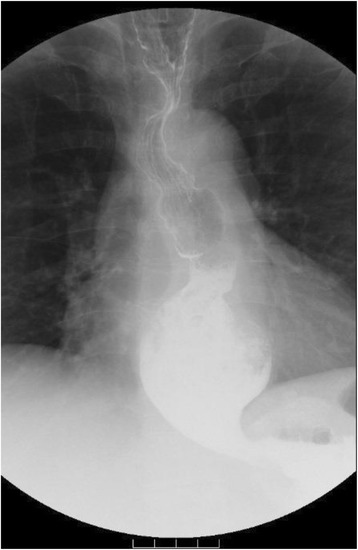
The maximum transverse diameter of the esophagus by esophagography was found to be 63 mm and was classified as the *straight shape*

**Fig. 8:**
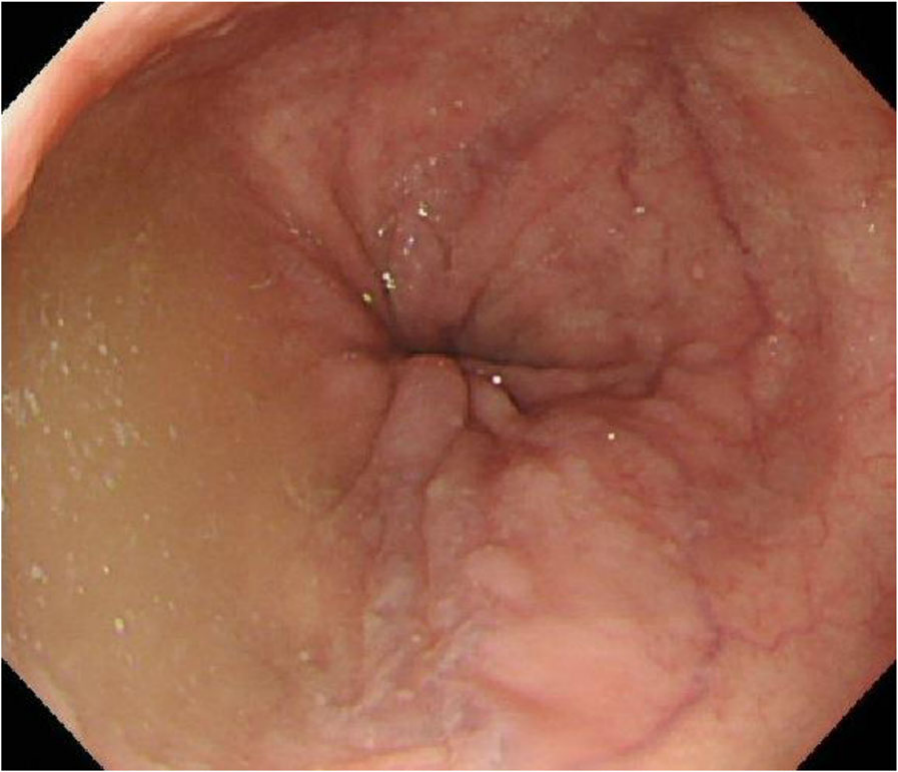
The esophageal lumen was distended, in which accumulation of saliva was observed

The case pertains to a woman in her sixties who is the mother of the patient in Case 1. She has been aware of dysphagia since approximately 50 years ago, but did not seek medical advice as the symptom did not affect her daily life. This time, she underwent a close examination because of the discovery of achalasia in her son. A definitive diagnosis of type 1 achalasia according to the Chicago classification was obtained by HRM (Fig. 5). The esophagus was found to be straight upon upper gastrointestinal series which corresponded with achalasia with a maximum transverse diameter of 60 mm (Fig. 6). Moreover, accumulation of saliva and the distended esophagus were observed upon upper gastrointestinal endoscopy (Fig. 7). The distended esophagus, liquid accumulation in the esophagus, and thickening of the esophageal wall were observed by CT (Fig. 8). In the same manner as Case 1, the serum ACTH and cortisol were within normal ranges, with no alacrima, muscular atrophy, or muscle weakness.

### Discussion

Familial achalasia is very rare. Allgrove syndrome may be referred to with respect to the relation between achalasia and the genes, which is an autosomal recessive disease with three signs, achalasia, alacrima, and adrenocortical insufficiency complicated by muscular atrophy and muscle weakness, and was first reported in 1978 by Allgrove et al. [4] The ALADIN gene has been identified as the responsible gene [5–8]. Ehrich et al. [9] reported that mild or moderate growth disorders and delayed psychomotor development are observed with this syndrome. Although mental deficiency was a complication in Case 1, no alacrima, adrenocortical insufficiency, or muscle weakness was observed, and although a genetic analysis had not been carried out, Allgrove syndrome was ruled out. A relation with Down’s syndrome has also been reported by Zárate et al. [10] who described that children suffering from Down’s syndrome are 200-fold more at risk for the onset of achalasia. Moreover, the authors compared 58 patients with Down’s syndrome with 38 healthy individuals and reported that the morbidity of gastric motility disorder was significantly higher in patients with Down’s syndrome, particularly with achalasia [11]. Although mental deficiency was a complication of the disease in Case 1, the subject did not have Down’s syndrome.

Occasionally, conditions similar to achalasia have been exhibited with malignant tumors, or vagotomy, which is referred to as secondary achalasia [12–15]. Gockel et al. [13] reported on the cause thereof, stating that these are observed as complications of malignant tumors at a rate of approximately 70% (primary: 53.9%, metastatic: 14.9%) and 11.9% following cardioplasty in distal esophageal and proximal gastric surgery. Nensey et al. [14] reported that from a total of 53 cases of secondary achalasia, gastric cancer (62%) was the most common as the cause thereof, followed by lung cancer (9%), malignant lymphoma (8%), pleural mesothelioma (5%), hepatocellular carcinoma (4%), and esophageal squamous cell carcinoma (4%), with the majority of mechanisms involving direct tumor invasion of the Auerbach nerve plexus or vagus nerve, along with destruction or consolidation of the esophago-gastric junction (EGJ) by the tumors. Moreover, Tucker et al. [15] reported that there are three signs acting as the standard for suspecting secondary achalasia, including (1) onset at age 55 years of age or older; (2) short duration of symptoms with the disease (within 1 year); and (3) prominent weight loss. Due to the fact that he was free of any of the three signs, the proximal margin of the tumor kept at distance from the EGJ by approximately 30 mm, and from the fact that images of esophagography was compatible with typical achalasia, Case 1 was determined as primary achalasia and was diagnosed to have concomitant gastric GIST with achalasia as a concomitant disease. Case 2 did not have diseases causing secondary achalasia by upper gastrointestinal endoscopy or CT scan, leading to a diagnosis of primary achalasia.

Generally, GIST is defined as abnormal proliferation of interstitial cells of Cajal (ICC); however, in recent years, the relationship between ICC and achalasia has also been reported. Killic et al. reported that ICC of 9 patients with achalasia were evaluated and increased in the achalasia group [16]. On the other hand, Hoshino et al. reported that there was no difference compared with 62 patients with achalasia and 10 cases of control group [3]. In this way, there is no consensus in the relationship between ICC and achalasia at present.

Few reports were found as a result of searching PubMed with “familial achalasia” as the keyword; however, there have been no reports to date in which esophageal manometry was carried out to establish a definitive diagnosis of achalasia, making this to the best of our knowledge the first report of objective diagnosis. Moreover, in view of the morbidity, achalasia in parent and child is very rare and genetic factors may play a role in the pathogenesis of achalasia.

## Conclusions

We hereby report on a very rare case of familial achalasia that we experienced which may suggest a genetic element in the onset of achalasia, and reviewed the literature.

## Abbreviations

CT: Computed tomography
GIST: Gastrointestinal stromal tumor
HRM: High-resolution manometry
LES: Lower esophageal sphincter.
